# Histone FRET reports the spatial heterogeneity in nanoscale chromatin architecture that is imparted by the epigenetic landscape at the level of single foci in an intact cell nucleus

**DOI:** 10.1007/s00412-024-00815-z

**Published:** 2024-01-24

**Authors:** Zhen Liang, Ashleigh Solano, Jieqiong Lou, Elizabeth Hinde

**Affiliations:** 1 Department of Biochemistry and Pharmacology, University of Melbourne, Melbourne, VIC Australia; 2 Cancer and RNA Laboratory, St. Vincent’s Institute of Medical Research, Melbourne, VIC Australia; 3 Department of Medicine, Melbourne Medical School, St Vincent’s Hospital, University of Melbourne, Melbourne, VIC Australia; 4 School of Physics, University of Melbourne, Melbourne, VIC Australia

**Keywords:** Chromatin, Histone modification, Förster resonance energy transfer, Fluorescence lifetime

## Abstract

Genome sequencing has identified hundreds of histone post-translational modifications (PTMs) that define an open or compact chromatin nanostructure at the level of nucleosome proximity, and therefore serve as activators or repressors of gene expression. Direct observation of this epigenetic mode of transcriptional regulation in an intact single nucleus, is however, a complex task. This is because despite the development of fluorescent probes that enable observation of specific histone PTMs and chromatin density, the changes in nucleosome proximity regulating gene expression occur on a spatial scale well below the diffraction limit of optical microscopy. In recent work, to address this research gap, we demonstrated that the phasor approach to fluorescence lifetime imaging microscopy (FLIM) of Förster resonance energy transfer (FRET) between fluorescently labelled histones core to the nucleosome, is a readout of chromatin nanostructure that can be multiplexed with immunofluorescence (IF) against specific histone PTMs. Here from application of this methodology to gold standard gene activators (H3K4Me3 and H3K9Ac) versus repressors (e.g., H3K9Me3 and H3K27Me), we find that while on average these histone marks do impart an open versus compact chromatin nanostructure, at the level of single chromatin foci, there is significant spatial heterogeneity. Collectively this study illustrates the importance of studying the epigenetic landscape as a function of space within intact nuclear architecture and opens the door for the study of chromatin foci sub-populations defined by combinations of histone marks, as is seen in the context of bivalent chromatin.

**Supplementary Information:**

The online version contains supplementary material available at 10.1007/s00412-024-00815-z.

## Introduction

Inside the nucleus of every human cell there is approximately 2 m of DNA wrapped around histone proteins to form a string of nucleosomes, which are then folded into a three-dimensional (3D) network called chromatin (Bonev and Cavalli 2016; Luger et al. 2012). Packaging of DNA into chromatin enables the entire human genome to be compacted into a highly organised structure that globally is small enough to occupy the $\sim$ 10 µm diameter nuclear volume, and locally is sufficiently dynamic to modulate DNA template access toward the proteins that read, copy, and repair genetic information (Lai and Pugh 2017). The feature of local chromatin structure that controls DNA template access is nucleosome proximity and it is regulated by histone post-translational modifications (PTMs) (Martire and Banaszynski 2020). Histone modifying enzymes write PTMs into the histone tails protruding from each nucleosome core, and genomic sequencing methods alongside in vitro biochemical assays, have demonstrated that their presence within chromatin results in an increase or decrease in the spacing between nucleosomes (Buchwalter et al. 2019). This epigenetically regulated effect, which activates or represses transcription of the underlying DNA, is however, difficult to directly observe in the context of an intact cell nucleus. This is because the changes in nucleosome spacing that are taking place occur on a scale of $\sim$ 2–50 nm and are well below the diffraction limit of optical microscopy (Ou, et al. 2017; Lakadamyali and Cosma 2020; Ricci et al. 2015; Oneto et al. 2019). Thus, there is significant interest in the transcription field for development of microscopy methods that can get around this technical hurdle and enable direct observation of the impact specific histone PTMs have on chromatin network architecture at the level of nucleosome proximity.

One way to get around the diffraction limit of optical microscopy and directly probe chromatin network architecture at the level of nucleosome proximity within an intact cell nucleus is to spatially map Förster resonance energy transfer (FRET) between fluorescently labelled nucleosomes throughout each pixel of a diffraction limited image (Lleres et al. 2009; Lleres et al. 2017; Pelicci et al. 2019). FRET is an optical phenomenon where a donor fluorescent molecule upon excitation transfers its absorbed energy to a nearby spectrally compatible acceptor fluorescent molecule (Clegg 1995). The efficiency of this non-radiative dipole–dipole interaction depends on distance across a scale of $\sim$ 1–10 nm and it results in the donor fluorescence lifetime being increasingly quenched with reduced donor–acceptor proximity (Hinde et al. 2012). Thus, in the context of a chromatin network where nucleosomes are globally labelled with donor and acceptor fluorophores (e.g., eGFP and mCherry) that are individually tagged to a core histone (e.g., histone H2B), detection of histone FRET serves as a molecular ruler of local nucleosome proximity (Lleres et al. 2009, 2017; Pelicci et al. 2019). In recent work we demonstrated that the phasor approach to fluorescence lifetime imaging microscopy (FLIM) is a robust method for detection of histone FRET that can spatially map this nanoscale readout of chromatin compaction throughout a living cell in the presence of broad spectrum cellular autofluorescence (Lou et al. 2019; Liang et al. 2020) and a fixed cell in the presence of fluorescent dyes routinely employed by immunofluorescence (IF) (Lou et al. 2021). Thus, phasor FLIM of histone FRET has the potential to enable direct observation of the nucleosome spacing defined by specific histone PTMs throughout intact nucleus architecture, in the instance these epigenetic marks can be specifically labelled by IF.

In recent decades, IF has enabled hundreds of histone post-translational modifications classified within sequencing data as gene activators and or gene repressors associated with open and or compact chromatin, to be spatially mapped throughout the nucleoplasm of a fixed cell. For example, development of commercially available primary antibodies against gold standard gene activating histone marks such as histone H3 tri-methylation of lysine 4 (H3K4Me3) and acetylation of lysine 9 (H3K9Ac) versus gene repressing histone marks such as histone H3 tri-methylation of lysine 9 (H3K9Me3) and lysine 27 (H3K27Me3) coupled with fluorescent secondary antibodies (e.g., labelled with Alexa Fluor dyes) has enabled microscopy studies that demonstrate transcription of gene-rich chromatin occurs throughout the central part of the nucleoplasm while repression of gene-poor chromatin is largely maintained at the nuclear periphery (Buchwalter et al. 2019). Thus, by combining this fluorescent readout of the epigenetic landscape with phasor FLIM of histone FRET there is the possibility to not only extract the spatial distribution of a histone PTMs within an intact nucleus, but also finally, the nanoscale chromatin compaction status they colocalise with at the level of nucleosome proximity.

Here to validate this technological capacity in the context of the epigenetic landscape, we first demonstrate that phasor FLIM of histone FRET multiplexed with IF does statistically report an open versus compact chromatin nanostructure for histone PTMs identified by genomic sequencing as gene activators and repressors, at the single cell level. Then we employ this three-colour experiment (histone FRET pair and IF label) to explore within a single cell the spatial heterogeneity in chromatin nanostructure that underlies these opening and compacting events at the level of single histone PTM foci. Finally, we extend this multiplexed method to a four-colour experiment (addition of one IF label) that incorporates RNA polymerase 2 (RNAP2) localisation, to investigate the interplay between the epigenetic landscape, nanoscale chromatin structure and transcription. Collectively these experiments reveal a surprising level of heterogeneity in the chromatin compaction status defined by gold standard histone PTMs at not only the single cell level, but also spatially throughout a single nucleus, while opening the door to investigate more ambiguous histone PTMs and their combinatorial action in the context of bivalent chromatin (Kumar et al. 2021), which can both activate and repress gene transcription (Hubner et al. 2013).

## Results

**Fig. 1:**
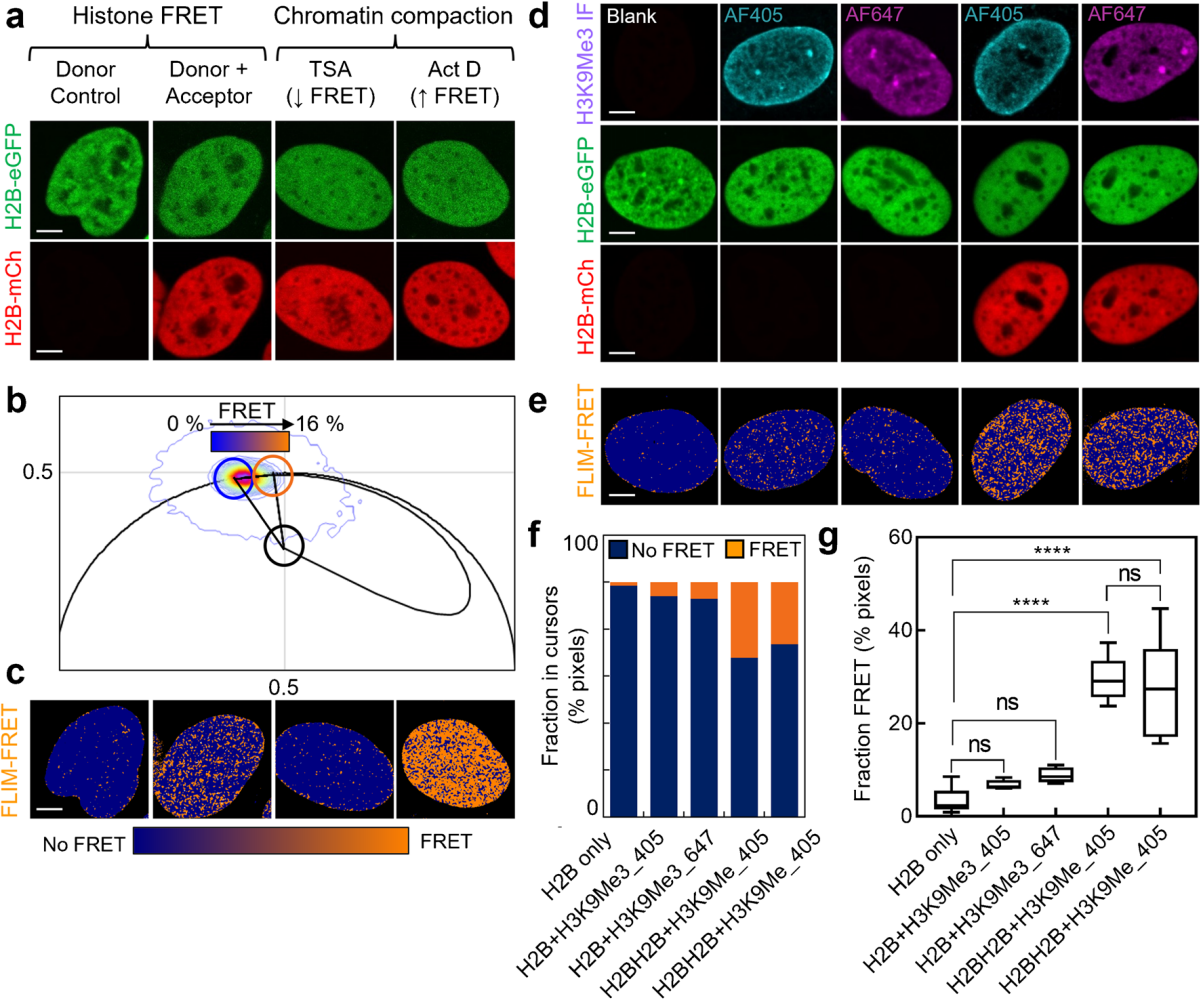
Phasor FLIM of histone FRET is compatible with cell fixation and IF. **a**. Fixed HeLa nuclei expressing H2B-eGFP in the absence versus presence of H2B-mCh and chromatin opening (Trichostatin A) versus compacting (Actinoymcin D) drug treatments. **b-c**. FLIM data recorded in the H2B-eGFP channel of the experiments presented in (a) transformed into a phasor plot (b) alongside maps of histone FRET (c). A theoretical FRET trajectory is superimposed (black curve) over the phasor plot (b) that extends from the unquenched donor lifetime (blue cursor) (2.5 ns). This FRET trajectory enables characterisation of the histone FRET efficiency as 16% (orange cursor) (2.1 ns) and definition of phasor cursors that spatially map no FRET (blue pixel) versus histone FRET (orange pixels) (c). **d**. Fixed HeLa nuclei expressing H2B-eGFP in the absence versus presence of H2B-mCH and IF against H3K9Me3 labelled with Alexa AF405 or AF647. **e**. Histone FRET maps derived from phasor analysis of FLIM data recorded in the H2B-eGFP channel of the experiments presented in (d). **f**. Quantification of the fraction of pixels in the no FRET (open chromatin) versus histone FRET (compact chromatin) state in the maps presented in (e). **g**. Quantification of the fraction of pixels in the histone FRET state (compact chromatin) across multiple cells in donor only, donor plus H3K9Me3-AF405 (donor + AF405), donor plus H3K9Me3-AF647 (donor + AF647), donor/acceptor plus H3K9Me3-AF405 (donor + Acc. + AF405), and donor/acceptor plus H3K9Me3-AF647 (donor + Acc. + AF647). Scale bars, 5 µm. N ≥ 4 cells, 1 biological replicate. The box and whisker plot in panel g shows the minimum, maximum, and sample median, with ns *P* > 0.05, ****P < 0.0001 (unpaired* t*-test)

**Multiplexing phasor FLIM of histone FRET with IF.** Visualisation of a histone PTM’s spatial distribution throughout intact nuclear architecture by IF requires: (1) cell fixation and (2) incubation with secondary IF antibodies conjugated to fluorescent dyes. Both these sample preparation steps have the potential to disrupt phasor FLIM of histone FRET. Thus, here we first aimed to demonstrate that histone FRET remains a quantitative readout of nanoscale chromatin compaction in fixed cells (Fig. 1a-c), and then identify IF labels that do not interfere with this photophysical phenomenon (Fig. 1d-f). To do so we first transiently transfected HeLa cells with H2B-eGFP (donor) in the absence (donor control) versus presence of H2B-mCH (acceptor) and treated samples containing both donor and acceptor (i.e., histone FRET experiment) with drugs known to loosen (Trichostatin A, TSA) or condense (Actinomycin D, Act D) chromatin (i.e., reduce or increase histone FRET) (Baum et al. 2014; Bensaude 2011) (Fig. 1a). Then in fixed and PBS washed HeLa nuclei expressing the donor control versus histone FRET experiments, we acquired FLIM data in the H2B-eGFP channel where quenching of the donor lifetime in the presence of H2B-mCh reports histone FRET. Quantification of these donor control and histone FRET experiments by the phasor approach to FLIM analysis (Methods) enabled definition of a phasor-based palette that extended from no histone FRET ($\sim$ 2.5 ns, unquenched H2B-eGFP) to 16% histone FRET ($\sim$ 2.1 ns, quenched H2B-eGFP) (Fig. 1b), which spatially maps and reports the expected fractional distribution of open (blue pixels) versus compact (orange pixels) chromatin throughout HeLa nuclei treated with TSA or Act D prior to fixation (Fig. 1c).

Upon establishing phasor FLIM of histone FRET as being compatible with cell fixation, we next explored the impact of fluorescent secondary antibodies that are a requirement for IF of the epigenetic landscape, on this assay’s capacity to report chromatin compaction. To do so we first performed IF against H3K9me3 labelled with no secondary antibody versus AF405 (blue, H3K9me3-AF405) or AF647 (far-red, H3K9me3-AF647), in fixed and PBS washed HeLa nuclei expressing H2B-eGFP (green, donor), in the absence versus presence of H2B-mCh (red, acceptor) (Fig. 1d). Then after an additional PBS wash, we acquired FLIM data in the H2B-eGFP channel, and from construction of pseudo-coloured maps of histone FRET (Fig. 1e) that report the fraction of open (no FRET) versus compact (FRET) chromatin (Fig. 1f), we investigated whether H3K9me3-AF405 or H3K9me3-AF647 quench this histone FRET donor’s fluorescence lifetime in the absence versus presence of H2B-mCh via an unwanted FRET interaction. Importantly, from quantitation of the fraction of pixels exhibiting FRET throughout multiple HeLa nuclei, we find neither H3K9Me3-AF405 or H3K9Me3-AF647 significantly quench the fluorescence lifetime of H2B-eGFP in the absence versus presence of H2B-mCh compared to histone FRET (Fig. 1g). Therefore, phasor FLIM of histone FRET can report nucleosome proximity in the presence of IF labelled histone PTMs.

**Fig. 2:**
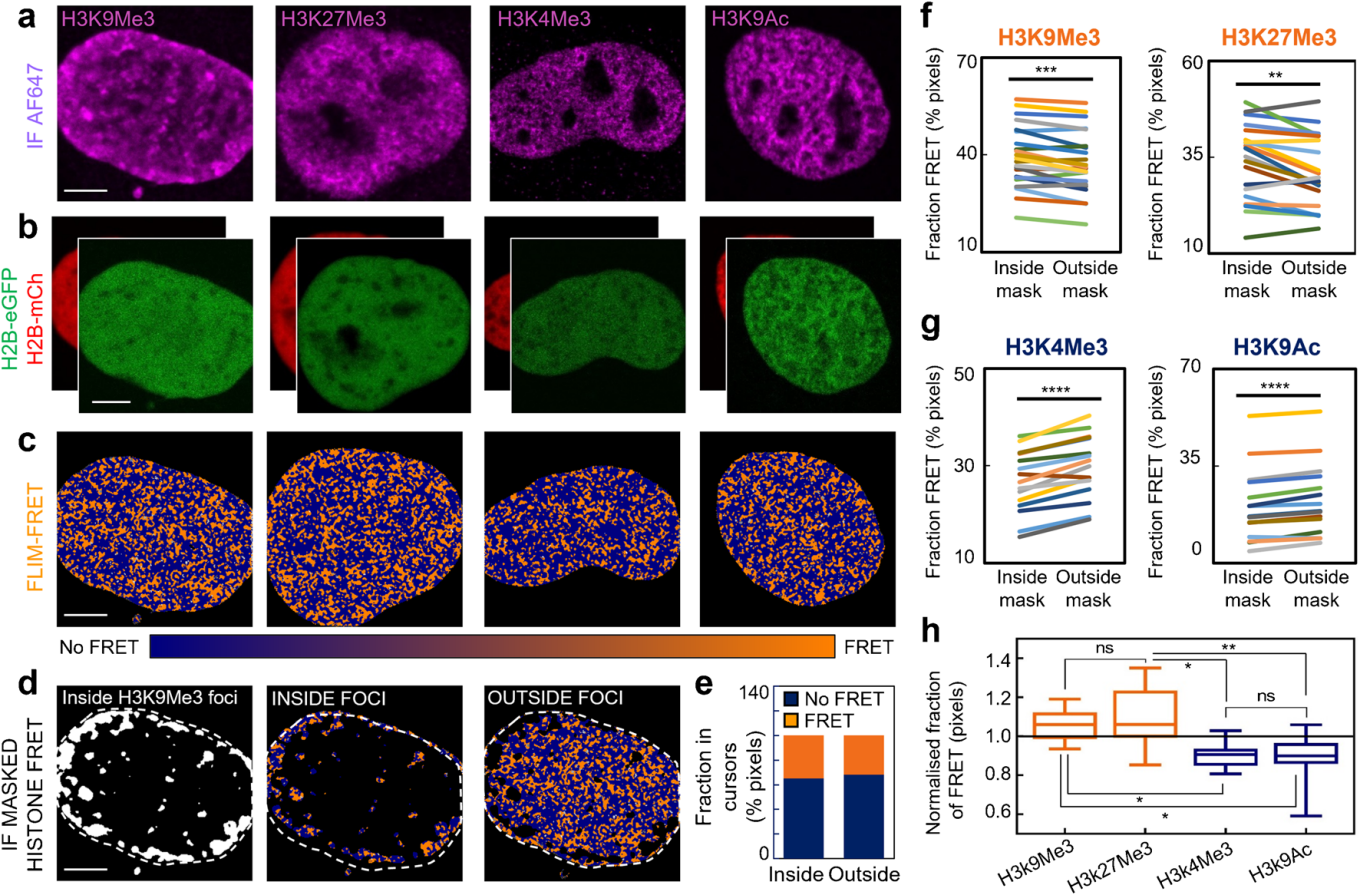
Phasor FLIM of histone FRET reports the nuclear wide chromatin nanostructure imparted by ‘open’ and ‘compact’ histone PTMs labelled by IF. **a-b**. HeLa nuclei fixed with IF (AF647) against H3K9Me3, H3K27Me3, H3K4Me3, and H3K9Ac (a) that are co-expressing the histone FRET pair H2B-eGFP and H2B-mCH (b). **c**. FLIM of the cells presented in panels (a)-(b) pseudo-coloured to report histone FRET (orange pixels) versus no-FRET (blue pixels). **d**. Mask based on H3K9Me3 IF signal presented in (a) (left panel) that selects pixels in the corresponding histone FRET map (c) that are inside (middle panel) versus outside (right panel) of high H3K9Me3 intensity regions. **e**. Fraction of pixels reporting FRET (compact chromatin) versus no FRET (open chromatin) within masked FLIM maps presented in panel d. **f-g**. Quantification of the fraction of histone FRET (compact chromatin) inside versus outside of high H3K9Me3, H3K27Me3, H3K4Me3, or H3K9Ac intensity regions. **h**. The ratio of histone FRET fraction (compact chromatin) inside versus outside of high intensity histone marker regions. Scale bars, 5 µm. *N* ≥ 15 cells, 3 biological replicates. In f-g **P < 0.01, ***P < 0.001, ****P < 0.0001 (paired *t*-test). The box and whisker plot in panel h shows the minimum, maximum, and sample median, with ns* P* > 0.05, *P < 0.05, **P < 0.01 (unpaired *t*-test).

**Phasor FLIM of histone FRET faithfully reports the chromatin nanostructure underpinning histone PTMs.** In the absence of intact nucleus architecture, advanced methods in genomic sequencing have identified several histone post-translational modifications as being strongly associated with either a compact (e.g., H3K9Me3 and H3K27me3) or open (e.g., H3K4me3 and H3K9Ac) chromatin structure that represses or activates transcription, respectively (Martire and Banaszynski 2020). Thus, here to investigate whether these key histone mark associations are preserved within intact nucleus architecture at the level of nucleosome proximity, we performed phasor FLIM analysis of histone FRET between H2B-eGFP and H2B-mCh co-expressed in fixed HeLa nuclei that have been labelled with IF against either H3K9Me3-AF647, H3K27Me3-AF647, H3K4Me3-AF647 or H3K9Ac-AF647 (Fig. 2a-b). This resulted in construction of pseudo-coloured maps of histone FRET (Fig. 2c), which upon subjection to a region of interest (ROI) analysis derived from an IF intensity mask (Fig. 2d), enabled the fraction of compact chromatin (i.e., histone FRET) associated with the high intensity regions of each histone mark (foci), versus the surrounding nucleoplasm, to be quantified (Fig. 2e). Strikingly, from application of this IF masked analysis of histone FRET to multiple HeLa nuclei we found that indeed the fraction of pixels exhibiting histone FRET inside the markers of ‘compact’ chromatin (H3K9Me3 and H3K27Me3) was statistically higher than the surrounding chromatin environment (Fig. 2f), while the fraction of pixels exhibiting histone FRET inside the markers of ‘open’ chromatin (H3K4Me3 and H3K9Ac) was statistically lower than the surrounding chromatin environment (Fig. 2g). This result can be summarised by normalising the fraction of pixels exhibiting histone FRET inside each epigenetic mark with respect to the surrounding nucleoplasm (i.e., chromatin compactors are > 1 while chromatin openers are < 1) (Fig. 2h). And although this result demonstrates phasor FLIM of histone FRET does faithfully report the expected shift in chromatin nanostructure underpinning key histone PTMs characterised by genome sequencing, the median value and range of the normalised histone FRET fractions calculated (Fig. 2h), also suggest a surprising level of heterogeneity in the chromatin compaction status defined by a histone PTM, at not only the single cell level, but also spatially throughout a single nucleus.

**Fig. 3:**
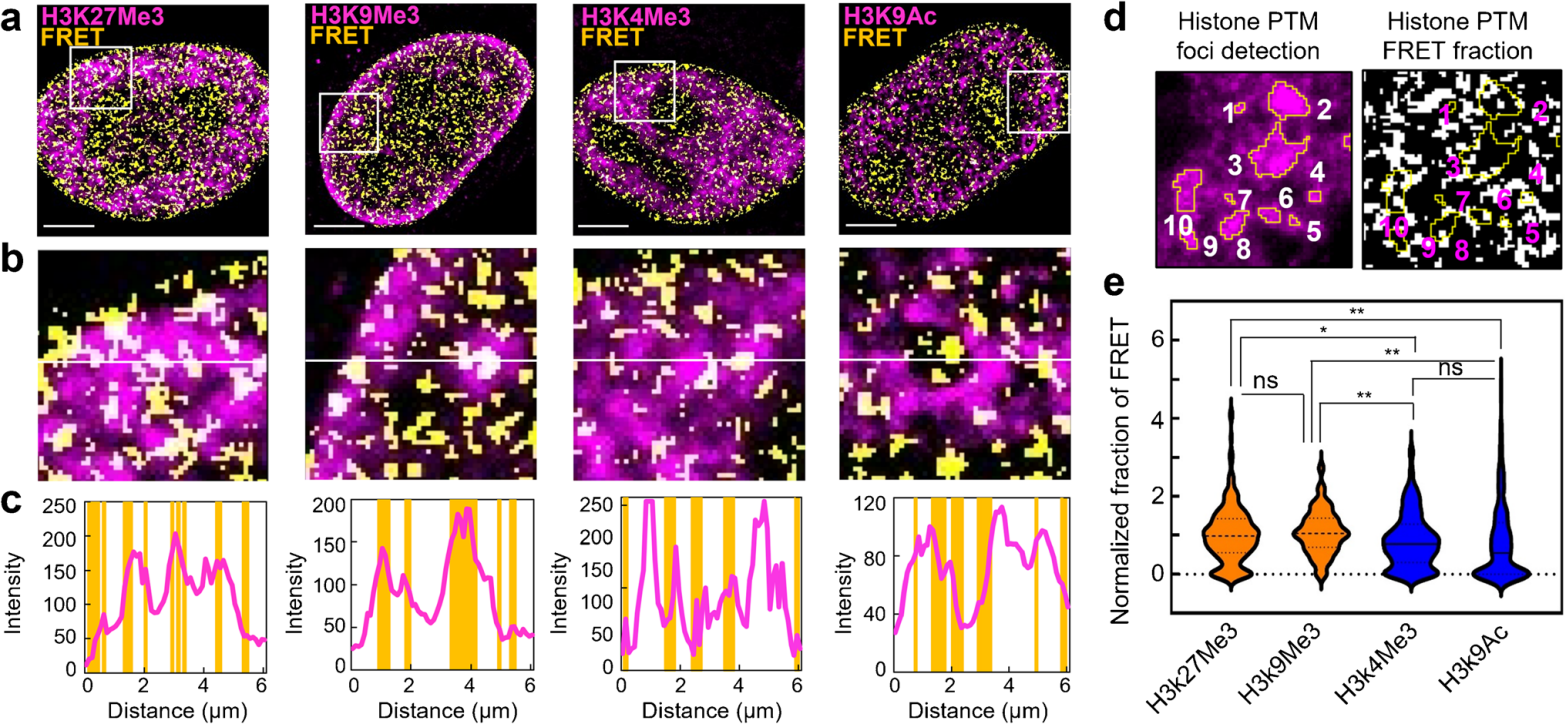
Phasor FLIM of histone FRET enables visualisation and quantification of the chromatin nanostructure imparted by a histone PTM at the single foci level.** a**. Intensity images of H3K27Me3, H3K9Me3, H3K4Me3, and H3K9Ac IF (magenta) merged with their corresponding binary chromatin compaction maps (yellow), which were generated via the workflow presented in Fig. 2. **b**. Zoomed in images of ROIs selected from the merged images presented in (a) (white square). **c**. Intensity of H3K27Me3, H3K9Me3, H3K4Me3, and H3K9Ac accumulation with respect to compact chromatin localisation along the central line within the selected ROIs in (b). **d**. An example ROI with individual histone PTM foci indexed (left) and the corresponding binary FRET map indexed based on foci detection (right). **e**. Quantification of the FRET pixel fraction in individual epigenetic foci normalized against FRET pixel fraction in the whole nucleus across multiple nuclei. Scale bars, 5 µm. N > 1000 foci from ≥ 10 cells, 3 biological replicates). The violin plot in panel e shows the sample median, 25% percentile and 75% percentile, with ns *P* > 0.05, *P < 0.05, **P < 0.01 (one way ANOVA)

**Phasor FLIM of histone FRET enables spatial heterogeneity in the chromatin nanostructure imparted by a histone PTM to be explored.** Phasor FLIM analysis of histone FRET coupled with IF has so far enabled the nuclear wide chromatin nanostructure associated with histone PTMs to be quantified at the single cell level. Next to visualise and investigate the degree of spatial heterogeneity in this chromatin nanostructure at the level of single histone PTM foci, we applied a line profile and particle analysis (Fig. 3) to the phasor FLIM of histone FRET experiments performed in HeLa, which were labelled with IF against H3K9Me3, H3K27Me3, H3K4Me3 and H3K9Ac (Fig. 2). To do this, we overlaid each IF intensity mask of a histone PTM with its corresponding map of histone FRET only (compact chromatin) (Fig. 3a), and then compared histone PTM accumulation with compact chromatin localisation along a line across a selected ROI (Fig. 3b-c). This analysis revealed that H3K9Me3 foci predominantly correlated with compact chromatin and H3K4Me3 foci predominantly anti-correlated with compact chromatin, while H3K27Me3 and H3K9Ac foci co-localised with both compact and open chromatin to an equivalent degree (Fig. 3c). In order to statistically quantify this heterogeneity in histone PTM chromatin nanostructure, we next assigned a unique particle number to each histone PTM focus identified (Fig. 3d, left) and then calculated the normalised fraction of histone FRET within each particle’s area, with respect to the fraction of pixels exhibiting histone FRET throughout the cell nucleoplasm (Fig. 3d, right). When applied to multiple histone PTM foci (Fig. 3e), this analysis confirmed that there is significant heterogeneity in the chromatin nanostructure imparted by histone PTMs classified as chromatin ‘compactors’ (i.e., H3K9Me3 and H3K27Me3) versus chromatin ‘openers’ (i.e., H3K4Me3 and H3K9Ac). For example, although H3K9Ac foci statistically co-localise with chromatin that is more open than the surrounding nucleoplasm, there are H3K9Ac foci that exhibit up to ~ fivefold more histone FRET (compaction) than the nucleoplasm. And in contrast, while H3K27Me3 foci statistically co-localise with chromatin that is more compact than the surrounding nucleoplasm, there are H3K27Me3 foci that exhibit no FRET (open chromatin). Thus, phasor FLIM of histone FRET coupled with IF has the potential to enable study of chromatin foci sub-populations defined by a specific histone PTM.

**Fig. 4:**
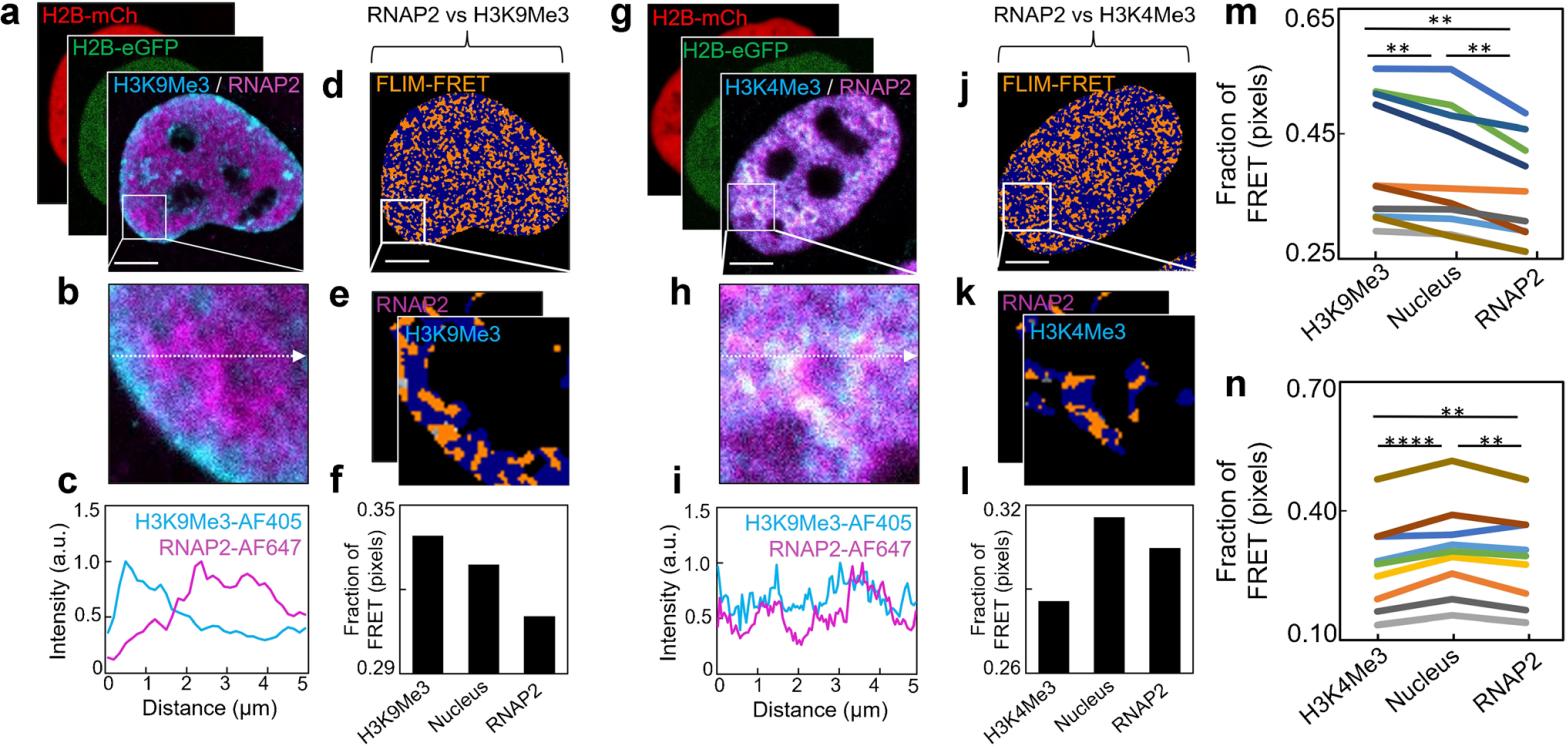
RNAP2 is associated with open chromatin. **a-b**. HeLa nucleus co-expressing the histone FRET pair H2B-eGFP and H2B-mCH that has been fixed with IF against H3K9Me3 and RNAP2 (a) and a ROI (b) selected for line and particle analysis. **c**. Line profile from the ROI in (b) showing H3K9me3 and RNAP2 anti-colocalise. **d-e**. FLIM of the cell presented in panel (a)-(b) pseudo-coloured to report histone FRET (orange pixels) versus non-FRET (blue pixels) (d) with a mask applied to the ROI (e) that associates with H3K9me3 versus RNAP2 intensity. **f**. Fraction of pixels reporting FRET (compact chromatin) in high H3K9Me3 intensity region, versus whole nucleus and high RNAP2 intensity region. **g-h**. HeLa nucleus co-expressing the histone FRET pair H2B-eGFP and H2B-mCH that has been fixed with IF against H3K4Me3 and RNAP2 (g) and a ROI (h) selected for line and particle analysis. **i**. Line profile from the ROI in (h) showing H3K4me3 and RNAP2 colocalise. **j-k**. FLIM of the cell presented in panel (g)-(h) pseudo-coloured to report histone FRET (orange pixels) versus non-FRET (blue pixels) (j) with a mask applied to the ROI (k) that associates with H3K4me3 versus RNAP2 intensity. **l**. Fraction of pixels reporting FRET (compact chromatin) in high H3K4Me3 intensity region, versus whole nucleus and high RNAP2 intensity region. **m–n**. Quantification of the fraction of histone FRET (compact chromatin) inside high H3K9Me3, total nucleus, and high RNAP2 intensity regions (m), versus inside high H3K4Me3, total nucleus, and high RNAP2 intensity regions (n) across multiple nuclei. Scale bars, 5 µm. *N* ≥ 10 cells, 2 biological replicates. **P < 0.01, ****P < 0.0001 (paired *t*-test)

**Phasor FLIM of histone FRET enables the interplay between the epigenetic landscape, chromatin nanostructure and transcription to be visualised within an intact nucleus.** RNAP2 transcribes all protein-coding genes in eukaryotic genomes and therefore is a good biomarker of where inside an intact nucleus transcription is occurring. Thus, to demonstrate how phasor FLIM of histone FRET multiplexed with IF can directly probe the interplay between histone PTMs known to associate with compact versus open chromatin structure and transcriptional activity, here in fixed HeLa co-expressing H2B-eGFP and H2B-mCh, we performed IF against RNAP2-AF647 alongside H3K9Me3-AF405 (Fig. 4a-f) versus H3K4me3-AF405 (Fig. 4g-l). As expected, H3K9Me3-AF405 that is a marker of gene repression, anti-colocalises with RNAP2-AF647 (Fig. 4c) and is associated with a significantly higher fraction of histone FRET than this biomarker of transcription, as well as the surrounding nucleoplasm (Fig. 4d-f and Fig. 4m). While in direct contrast, H3K4me3-AF405 that is a marker of gene activation, co-localises with RNAP2-AF647 (Fig. 4i) and is associated with a significantly lower fraction of histone FRET than this biomarker of transcription as well as the surrounding nucleoplasm (Fig. 4j-l and Fig. 4n). Thus collectively, this result (Fig. 4m-n) demonstrates that transcription does indeed prefer an open chromatin environment underpinned by an increase in nucleosome spacing, and this chromatin nanostructure is defined by an epigenetic landscape within an intact nucleus that agrees with genomic sequencing data. Thus, phasor FLIM of histone FRET multiplexed with IF against RNAP2 and histone PTMs offers an opportunity for cell biologists to investigate the chromatin nanostructure of more ambiguous histone post-translational modifications, with the added advantage of sensitivity toward spatial as well as cell to cell heterogeneity.

## Discussion

In this study we multiplexed phasor FLIM of histone FRET with IF against histone PTMs and demonstrated that this technology has sufficient spatial resolution to correlate the epigenetic landscape of an intact nucleus with chromatin nanostructure on a scale relevant to transcription. By focusing on gold standard histone PTMs identified as gene activators or repressors by genome sequencing (Lai and Pugh 2017; Martire and Banaszynski 2020; Buchwalter et al. 2019), and then confirming their association or dissociation with RNAP2 versus an open or compact chromatin structure at the level of nucleosome proximity, we validated this technical approach as a reliable means for researchers to explore the interplay between more ambiguous histone marks and gene expression. A notable advantage of phasor FLIM of histone FRET being coupled with histone PTM IF, when compared to advanced genomic sequencing methods such as chromosome conformation capture (3C), or related technologies such as Hi-C (Jerkovic and Cavalli 2021), is its capacity to uncover and explore the spatial heterogeneity in chromatin nanostructure imparted by a specific epigenetic signature. For example, upon investigating the chromatin nanostructure underpinning histone PTMs traditionally associated with gene repression via histone FRET, it became evident that although both H3K9me3 and H3K27me3 do statistically co-localise with a more compact chromatin structure, in the case of H3K27me3, this result was underpinned by significant heterogeneity, and several examples of H3K27me3 foci co-localising with open chromatin. This result likely reflects the fact that while H3K27me3 is a mark of facultative heterochromatin (Trojer and Reinberg 2007), this histone PTM is also reported to be: (1) a temporary repressive mark against transcription, unlike H3K9me3 that provides permanent repression, and (2) 30% associated with transcriptionally active euchromatin, in contrast to 3% for H3K9me3 (Becker, et al. 2017). Thus this analytical capacity of histone FRET could be invaluable when investigating the chromatin nanostructure of histone specific marks involved in both gene activation and repression (Lai and Pugh 2017; Martire and Banaszynski 2020; Buchwalter et al. 2019), or the cooperative impact of a histone code underpinned by multiple specific marks, as is seen in the context of bivalent chromatin (Kumar et al. 2021; Hubner et al. 2013). Future efforts will be devoted toward development of new labelling strategies for the core histone FRET analysis that enable exploration of different higher order chromatin structures regulated by specific histone marks beyond nucleosome proximity and their impact on gene expression.

## Methods

**Cell culture, transient transfection, and immunofluorescence.** HeLa cells were grown in Dulbecco’s modified Eagle’s medium (Lonza) supplemented with 10% bovine growth serum (Gibco), 1 × Pen-Strep (Lonza) at 37 °C in 5% CO_2_ and then plated onto 35 mm glass-bottom dishes. After 24 h, plated HeLa cells were transiently transfected with the histone FRET pair H2B-eGFP and H2B-mCherry via use of Lipofectamine 3000 according to the manufacturer’s protocol. After a further 24 h, the transfected HeLa cells were fixed with 4% paraformaldehyde for 15 min, permeabilized with 1 mg/ml Triton X-100 for 15 min at room temperature and blocked with 1% bovine serum albumin for 30 min. Three rounds of washing with phosphate-buffered saline (PBS) were performed in between each of these fixation steps. Next, the fixed transfected HeLa cells were subjected to IF against one or two of the following histone post-translational modifications: H3K9Me3 (Ab8898, Abcam, H3K4Me3 (Ab8580, Abcam), H3K27Me3 (Abcam) and H3K90Ac (Abcam); alongside RNA polymerase II (RNAP2) (Abcam) in some instances. For both one and two-colour IF sample preparations, the transfected and fixed HeLa cells were incubated with the primary antibody or antibody pair (1:200) overnight at 4 °C and then the secondary antibody or antibody pair labelled with Alexa Fluor 405 (AF405) and or Alexa Fluor 647 (AF647) for 1 h at room temperature. The three rounds of washing steps with PBS were also performed in between each of these IF steps. In general, PBS washing not only was critical for fixation and IF but also counteracted a chemically induced shift in the fluorescence lifetime of H2B-eGFP that was unrelated to histone FRET (Fig. S1).

**Confocal laser scanning microscopy and FLIM data acquisition.** All fixed cell fluorescence intensity and lifetime microscopy measurements were performed on an Olympus FV3000 laser scanning microscope coupled to a 488 nm pulsed laser operated at 80 MHz and an ISS A320 Fast FLIM box. A 60 × water immersion objective 1.2 NA was used for all experiments and the cells were imaged at room temperature. First, multi-channel intensity images (512 × 512 pixel frame size, 12.5 µs/pixel, 45 nm / pixel) were acquired of each selected HeLa nucleus to: (1) verify the presence of the histone FRET pair (H2B-eGFP and H2B-mCh) with an acceptor-donor ratio > 1 and (2) record the spatial localisation of AF405 and or AF647 marked histone PTMs, in some instances alongside RNAP2. This involved sequential imaging of a two-phase light path in the Olympus FluoView software. The first phase was set up to image H2B-eGFP and H2B-mCh via use of solid-state laser diodes operating at 488 and 561 nm, respectively, with the resulting signal being directed through a 405/488/561/6033 dichroic mirror to two internal GaAsP photomultiplier detectors set to collect 500–540 nm and 600–700 nm. The second phase was set up to image AF405 and or AF647 via use of solid-state laser diodes operating at 405 and 633 nm, respectively, with the resulting signal being directed through a 405/488/561/633 dichroic mirror to two internal GaAsP photomultiplier detectors set to collect 420–460 nm and 600–700 nm. Then in each HeLa nucleus selected, a FLIM map of H2B-eGFP was imaged within the same field of view (256 × 256-pixel frame size, 20 µs/pixel, 90 nm/pixel, 20 frame integration) using the ISS VistaVision software. This involved excitation of H2B-eGFP with the external pulsed 488 nm laser (80 MHz) and the resulting signal being directed through a 405/488/561/633 dichroic mirror to an external photomultiplier detector (H7422P-40 of Hamamatsu) that was fitted with a 520/50 nm bandwidth filter. The donor signal in each pixel was then subsequently processed by the ISS A320 FastFLIM box data acquisition card to report the fluorescence lifetime of H2B-eGFP. All FLIM data were pre-calibrated against fluorescein at pH 9 which has a single exponential lifetime of 4.04 ns.

**FLIM-FRET analysis.** The fluorescence decay recorded in each pixel of an acquired FLIM image was quantified by the phasor approach to lifetime analysis. As described in previously published papers (Digman et al. 2008; Ranjit et al. 2018), this results in each pixel of a FLIM image giving rise to a single point (phasor) in the phasor plot, which when used in the reciprocal mode enables each point in the phasor plot to be mapped to each pixel of the FLIM image. Since phasors follow simple vector algebra, it is possible to determine the fractional contribution of two or more independent molecular species coexisting in the same pixel. For example, in the case of two independent species, all possible weightings give a phasor distribution along a linear trajectory that joins the phasors of the individual species in pure form. While in the case of a FRET experiment, where the lifetime of the donor molecule is changed upon interaction with an acceptor molecule, the realization of all possible phasors quenched with different efficiencies describes a curved FRET trajectory in the phasor plot that follows the classical definition of FRET efficiency (Hinde et al. 2012).

In the context of the histone FRET experiments presented, the phasor coordinates (g and s) of the unquenched donor (H2B-eGFP) and background (cellular autofluorescence) were first determined independently in fixed HeLa cells transfected versus un-transfected with H2B-eGFP. This enabled definition of a baseline from which a FRET trajectory could be extrapolated and then used to determine the dynamic range of FRET efficiencies that describe chromatin nanostructure in the HeLa system. From superimposition of this FRET trajectory with the combined phasor distribution measured for H2B-eGFP in fixed HeLa cells co-transfected with H2B-mCh, we find the HeLa chromatin network to exhibit chromatin compaction states that range from 0 to 16% in FRET efficiency. This corresponds to a shift in the H2B-eGFP donor lifetime from approximately 2.5 ns to 2.1 ns. We therefore defined two cursors centered at these phasor coordinates to spatially map where chromatin is open (blue cursor) versus compact (orange cursor) throughout a FLIM data acquisition in a fixed HeLa nucleus and quantify overall chromatin network compaction based on the fraction of pixels exhibiting histone FRET (i.e., number of pixels in 2.1 ns orange cursor / (number of pixels in 2.5 ns blue cursor + number of pixels in 2.1 ns orange cursor). All FLIM-FRET quantification was performed in the SimFCS software developed at the Laboratory for Fluorescence Dynamics and a Matlab code specifically designed to mask FLIM data according to an IF signal, which enabled calculation of the chromatin nanostructure defined by histone PTMs.

**IF mask analysis of histone FRET**. To quantify the chromatin nanostructure associated with different histone PTMs, we applied an intensity threshold mask based on H3K4Me3, H3K9Ac, H3K9Me3, and H3K27Me3 IF intensity images to FLIM maps pseudo-coloured according to histone FRET (compact chromatin) versus no FRET (open chromatin). This involved: (1) smoothing each HeLa nucleus’ IF image of histone PTM localisation with a 3 × 3 spatial median filter, (2) transforming this smoothed image into a binary mask based on an intensity threshold that was sufficiently harsh to reject non-specific IF staining but retain histone PTM foci (i.e., top 5% intensity pixels), (3) applying the IF-guided mask to its associated FLIM map pseudo-coloured according to histone FRET, and (4) quantification of the fraction of compact chromatin within (i.e., histone PTM foci) versus outside (i.e., nucleoplasm) the IF-guided mask in total and as a function of foci number.

### Supplementary Information

Below is the link to the electronic supplementary material.

Supplementary file1 (PDF 640 KB)

## Data Availability

The datasets generated during and/or analysed during the current study are available from the corresponding author on reasonable request.
