# Supplementary material for: Histone FRET reports the spatial heterogeneity in nanoscale chromatin architecture that is imparted by the epigenetic landscape at the level of single foci in an intact cell nucleus
Source: Chromosoma. 2024 Jan 24;133(1):5–14. doi: 10.1007/s00412-024-00815-z (PMC10904561; doi:10.1007/s00412-024-00815-z)
Supplement: Supplementary file 1 — Supplementary file1 (PDF 640 KB) [file 412_2024_815_MOESM1_ESM.pdf]

Supporting Information

Histone FRET reports the spatial heterogeneity in nanoscale chromatin architecture that is imparted by the epigenetic landscape at the level of single foci in an intact cell nucleus.

Zhen Liang[1,2,3*], Ashleigh Solano[4*], Jieqiong Lou[4*], and Elizabeth Hinde[1,4#].

1. Department of Biochemistry and Pharmacology, University of Melbourne, Melbourne, Victoria, Australia.
2. Cancer and RNA Laboratory, St. Vincent's Institute of Medical Research, Melbourne, Victoria, Australia.
3. Department of Medicine, Melbourne Medical School, St Vincent's Hospital, University of Melbourne, Melbourne, Victoria, Australia.
4. School of Physics, University of Melbourne, Melbourne, Victoria, Australia.

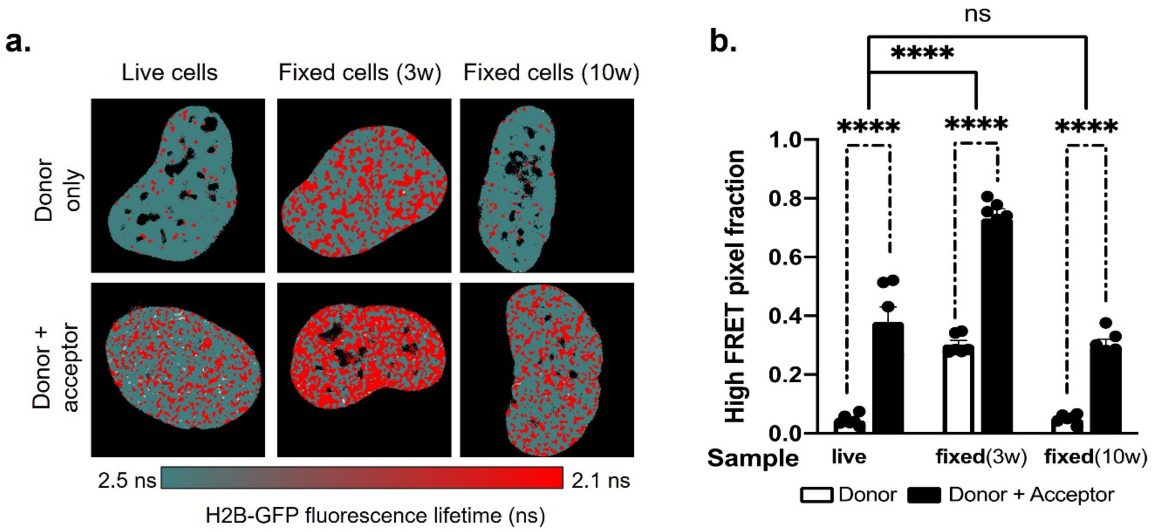

Figure S1. Extensive PBS washing during cell fixation is required to prevent a chemical quenching of H2B-eGFP's fluorescence lifetime (our readout of histone FRET). (a) Histone FRET maps derived from HeLa cells that are expressing only H2B-eGFP (i.e., donor control) (top row) versus H2B-eGFP and H2B-mCh (i.e., donor and acceptor) (bottom row) when they are alive (left column) versus after fixation with: (1) 3 PBS washing steps (3w) (middle column) or (2) 10 PBS washing steps (10 w) (right column). **(b)** Quantification of the fraction of histone FRET across multiple cells represented in (a) reveals fixation to quench the donor lifetime (i.e., donor control after 3w exhibits a significant histone FRET fraction) and extensive washing with PBS (i.e., 10 w) to reverse this artefact for histone FRET analysis.
